# The Politics of Risk: A Human Rights Paradigm for Children’s Environmental Health Research

**DOI:** 10.1289/ehp.9002

**Published:** 2006-08-14

**Authors:** Maura A. Ryan

**Affiliations:** Department of Theology, University of Notre Dame, Notre Dame, Indiana, USA

**Keywords:** environmental health, environmental policy, ethics, human rights, pediatric research

## Abstract

A human rights paradigm for environmental health research makes explicit the relationship between poor health and poverty, inequality, and social and political marginalization, and it aims at civic problem solving. In so doing, it incorporates support for community-based, participatory research and takes seriously the social responsibilities of researchers. For these reasons, a human rights approach may be better able than conventional bioethics to address the unique issues that arise in the context of pediatric environmental health research, particularly the place of environmental justice standards in research. At the same time, as illustrated by disagreements over the ethics of research into lead abatement methods, bringing a human rights paradigm to bear in the context of environmental health research requires resolving important tensions at its heart, particularly the inescapable tension between ethical ideals and political realities.

## The Politics of Risk

Recent debates over the conduct of children’s environmental health research have highlighted the importance of integrating environmental justice standards into the design, implementation, and evaluation of research paradigms (Pinder 2002). In addition to acknowledging the different vulnerability of children to the effects of environmental toxicants, environmental justice concerns recognize the structural conditions (e.g., race, income, housing options) that account for differential distribution of environmental health hazards across communities and potentially constrain not only the conditions for informed consent/assent but the prospects for research outcomes to result in health gains for at-risk children. A human rights paradigm for environmental health research takes the relationship between poor health and poverty, inequality, and social and political marginalization as its starting point and aims to develop capacities within communities. For these reasons, a human rights approach may be better equipped than conventional bioethics—which has developed largely within the clinical setting and which has tended to focus on the maximization of individual autonomy—to address the unique issues that arise in the context of public health research and therefore in the context of pediatric environmental health research. At the same time, as a brief look at debates over the ethics of lead abatement studies shows, invoking a human rights framework raises important questions—particularly how to balance practical limits and utopian goals.

## Public Health, Ethics, and Human Rights

Callahan and Jennings (2002) identify four characteristics of the setting for public health research that distinguish it from research ethics generally and that lend an affinity to the international human rights discourse that has developed since World War II and currently informs global health initiatives. First, health is considered a public or “common” good. Despite disagreements over the foundation and scope of human rights and the nature of a “right to health,” collaborative efforts to improve public health globally presuppose the importance of health for human dignity and human flourishing. In illuminating the links among health, human development, and the environment, environmental health research assumes the importance of a safe environment, not only for individual children but also for sustaining the human species (Landrigan et al. 2004). Public health research is oriented toward informing interventions, reforms, and policies that promote and protect population health. Thus it can be at odds with the general priorities of clinical research, particularly industry-sponsored clinical research, and with the bias of contemporary bioethics toward the promotion of individual autonomy.

Second, because public health measures aim at population health, communities rather than individuals are the primary locus for moral agency. Increased sensitivity to the differential burdens of environmental hazards across communities, as well as to the intersections of poverty, race, and environmental health, has highlighted the value of community-based participatory research (Weed and McKeown 2003). One implication of a community-based participatory approach to children’s environmental health research is greater attention to the ethical significance of choices about where to conduct research. Because environmental risks are differentially distributed across communities, the benefits of documenting environmental health risks—for example, in providing data for determining the best strategies for prevention and remediation—must be weighed against the possible harms to the community (e.g., the potential to discourage economic investment in the area). Active partnerships between academic researchers and community leaders recognize the importance of community input in the weighing process. In addition, children’s environmental health research can involve particularly vulnerable communities and thus can raise questions about the conditions for informed consent, the appropriateness of incentives to participate, the scope of obligations to report findings, and protections for privacy. For example, because low-income families and people of color are much more likely to live in substandard housing, research into the prevention and amelioration of lead poisoning and other housing-related diseases will disproportionately involve low-income and minority communities (Ryan and Farr 2002). To avoid the dangers of paternalism on the one hand and exploitation on the other hand, the appropriate role of the community extends not only to consultation with respect to study design and procedures for obtaining informed consent, but also participation in determining how research results will be used for its benefit (American Academy of Pediatrics 2004).

Third, a further implication of community-based, participatory research in pediatric environmental health is that it is “intentionally contextualized, rather than intentionally random” (Sharpe 2002). Research is oriented toward developing practical and feasible solutions to the self-identified needs of an affected or potentially affected community, rather than driven principally or exclusively by commercial or academic interests. To an interpretation of context, a human rights framework adds explicit acknowledgment of the social, economic, and political determinants of health and illness—specifically the relationship between poor health and poverty, inequality, and social and political marginalization (Sharpe 2002). Thus, poor health as a moral problem is assumed to result not only from “the behavior of certain disease organisms or particular individuals... but [from] institutional arrangements and prevailing structures of cultural attitudes and social power” (Callahan and Jennings 2002). The remedy for poor health outcomes is sought in “social rather than in specific biological interventions” (Sharpe 2002).

One consequence is that ethical considerations pertain not only to the conduct of research—whether conditions such as scientific merit, equipoise, and the duty to obtain informed consent are met—but to the aim of research—whether it is likely to contribute to appropriate and effective social interventions. This means that the complex relationship between the ethical and the political is both more visible and more integral for a human rights paradigm.

Fourth, politics mediates environmental health research in the obvious sense that political factors as well as social and economic factors shape the context in which the aims of research are defined, funding for research is appropriated, and research results are translated into health and environmental policy. In part, political climate determines whether policy makers and government agencies will be responsive to industry pressures to relax environmental protection standards or responsive to pressures from children’s health advocates to enact or strengthen measures that address the particular vulnerability of children to environmental toxicants and aim at preventing or reducing exposure (Mielke 2002). Political, social, and economic power and visibility also play a role in setting research priorities. Children’s environmental health research can suffer a three-way disadvantage in competition for research funding. First, as an arm of public health research it runs up against a long-standing tendency in U.S. health care to focus on end-stage interventions and to invest in cure over prevention. Second, the study of children and childhood has always been accorded a marginal place in the health, human, and social sciences (Berman 2003). This results partly from a prevailing assumption that childhood is a temporary, private, or domestic (vs. social) state as well as from a tendency to treat children as a homogeneous group, without attention to differences of race, class, or sex. Finally, the groups from which the children who are most at risk of exposure to environmental toxicants come are themselves in greater danger of marginalization in the setting of research priorities. For example, a 1999 Institute of Medicine study concluded that National Institutes of Health funding for cancer research targeting minority and medically underserved populations (which will include many at-risk children) was both inadequate and unequal in comparison to research targeting nonminority populations (Haynes and Smedley 1999). The vulnerability of public health research to political and economic forces is well illustrated by the uncertain fate of the National Children’s Study, the federally funded longitudinal, comprehensive study of the multiple environmental factors affecting children’s health and development initiated in 2000. Now in its preliminary phase, there is doubt about whether sufficient funding will be allocated in 2007 for the project to move ahead (National Children’s Study 2006).

More important, when we say that the relationship between the political and the ethical is more integral and more visible within a human rights paradigm than for a traditional research paradigm, we mean that the social responsibilities of scientists—particularly their role as advocates for reforms aimed at addressing the social and economic inequalities that are factors in environmental health—are primary rather than secondary concerns. Reiser and Bulger (1997) note three basic opportunities for scientists to exercise social responsibility: in decisions about whether to participate in a particular research project and how it should be conducted; in alerting society to possible benefits and harms of developments or discoveries; and in participating in the discussion of issues that arise from research and educating the public and public policy makers to make informed decisions. Since its development as a field, bioethics has been acutely sensitive to the first sense of scientific responsibility. Ethical requirements to obtain the voluntary consent of research subjects, to demonstrate the scientific merit of a proposed study, and to ensure the safety of volunteers are all means of expressing social responsibility in the design and conduct of research. All these requirements are fundamental in defining what constitutes ethical research in pediatric environmental health.

As noted before, a human rights paradigm makes explicit the relationship between poor health and poverty, inequality, and social and political marginalization and characterizes research aims in terms of effective social interventions. This has further implications for defining ethical choices concerning why and how environmental health research will be conducted. The ethical conduct of research aimed at identifying susceptibility to environmental hazards or evaluating proposed interventions within marginalized or particularly vulnerable communities may require special attention to existing obstacles to obtaining genuinely informed and voluntary consent (e.g., language barriers, literacy levels, or cultural patterns of deference to authority); therefore, the role of local knowledge and community consultation becomes particularly important. A delicate balance must be sought between the timely dissemination of data to interested or affected parties and the responsibility to provide accurate, clear and meaningful information (Ryan and Farr 2002). Investigation of housing-related environmental health risks, which will disproportionately target low-income and minority populations, raises important ethical questions about how to weigh risks associated with participation in environmental health research against risks already existing in the potential subject’s everyday home environment and what monitoring tools best signal timely evidence of unacceptable risk. For studies conducted in a “sea of risk,” where it is unlikely that any child will escape some exposure, Mushak (2002) has argued for the importance of serial biomonitoring of “adequate frequency and within a trend analysis.” In this context, “an unacceptable exposure profile for purposes of quantifying efficacy in the prevailing research protocol is not the ethically unacceptable achievement of a toxic exposure but an unacceptably rapid trend rate toward potentially toxic exposure.”

Attention to inequality or powerlessness is also important in the justification of research design and research settings. Lavery et al. (2003) argue that the justification for conducting research on populations with known exposure to environmental hazards, and the need to articulate the relationship between the ends of research and the interests of the community, become stronger as limits on subjects or potential subjects to reduce or avoid hazards becomes greater. At the very least, when a study group is completely or disproportionately composed of members of racially, socially, or economically vulnerable populations, it has to be asked “whether the same approach would have been taken with nonminority groups or with the highly educated or those with adequate incomes” (Phoenix 2002).

Precisely because the ends of research aim at effective social interventions, however, a human rights paradigm highlights the third sense of scientific responsibility above: the importance of participation in the political process and the role of public education in bringing about meaningful, comprehensive reforms. Public health has always relied on law as well as on education to enact strategies for prevention of disease or promotion of health. Pediatric environmental health research has played an important role in the call for child-protective legislation (e.g., the Food Quality Protection Act of 1996) and child-sensitive approaches to risk assessment (Landrigan et al. 2004). A human rights approach takes this notion of political participation further, however, calling for the contribution of information and professional power to efforts to secure equitable access for all persons to basic health care and a safe environment. A human rights paradigm for environmental health assumes that social and economic rights are as fundamental as civil and political rights, that the ends of health and environmental policy are as important for bioethics as the means, and that “research and critical assessment [of relative risks and their health consequences] are necessary but not sufficient” (Farmer 1999).

For this approach, ethics extends beyond attention to individual behavior or to the balance between protecting individual liberties and promoting public health. It “calls for discussions of ethics and health policy to be genuinely public or civic endeavors,” characterized by “meaningful participation, open deliberation, and civic problem solving and capacity building” (Callahan and Jennings 2002). A human rights approach brings both a critical and a practical dimension to public deliberation in bringing attention to historical patterns, cultural values, and prevailing structures of power and powerlessness as they contribute to or result in disparities in access to the benefits of scientific progress or unequal exposure to environmental health hazards. This means that there is an intimate, although by no means straightforward, relationship between the fact-finding goal of research and public advocacy. Insisting on an ethical relationship between fact finding and advocacy does not mean defending interventions by well-informed and well-intended elites on behalf of affected communities (Callahan and Jennings 2002). Rather, it means that research conducted in a vacuum, without strategic attention to those contributing social values, prevailing interests, and structural conditions underlying disease burden and the ability to avoid or address health hazards, risks leaving unaddressed the most significant moral issues for at-risk individuals and communities.

This is not to suggest that there is no role for research as documentation in a human rights paradigm. Sound, verifiable information is needed, for example, on the relative risks of lead exposure in one method of lead abatement versus another. As Ryan and Farr (2002) point out, “until rigorous research documented the dangers of conventional, dust-generating paint removal methods, many state and local regulations prescribed removal of lead-based paint by power sanding or open flame burning, activities now banned in federally assisted housing and by many jurisdictions.” The point is, rather, that children’s environmental health goals, principally the prevention of exposure to toxic levels of lead at the source, will not be achieved without critical attention to the broad spectrum of contributing factors: environmental regulations, public housing policy, the enforcement of safe housing and health codes, and the overall political will to address poverty, particularly as it relates to children of color (Ryan and Farr 2002). Because it is oriented toward problem solving and capacity building, the ultimate ethical and political challenge of a human rights approach is the development of the civic virtue of “pragmatic solidarity” (Farmer 1999). This means the willingness in public forums to use the power of science and medicine in the service of equity, to challenge prevailing structures of power and powerlessness in developing practical solutions to real-world problems, and to question the values and priorities which govern how resources will be used and distributed.

## Ethics, Realities, and Limits

Although I have argued here that a human rights paradigm incorporates a richer set of concerns that are relevant to children’s environmental health research than does a conventional bioethics paradigm, particularly concerns for environmental justice, no approach is free of limitations or growing edges. Some of the limitations of a human rights paradigm for children’s environmental health research have already been suggested—for example, the difficulty of reconciling the objective character of scientific research with an advocacy role for scientists, especially as it pertains to different kinds of research under the broad umbrella of pediatric environmental health. Discussions of the intersections of health, human rights, and the environment do not contain a clear roadmap for discerning what constitutes appropriate political participation for scientists. In addition, it is not obvious how to weigh what has been called “the unwelcome trade-offs between practicality and scientific rigor” that follow from the practical orientation of public health research (Ryan and Farr 2002). As in the case of housing-related environmental health risks, the fact cannot be avoided that “even the most accurate and precise hazard assessment protocols provide little or no benefit to communities at risk unless they are accessible, easy-to-use, and relatively low cost” (Ryan and Farr 2002). The most interesting question raised by arguments for a human rights paradigm in environmental health research is how to reconcile its fundamentally utopian goals with its pragmatic, problem-solving orientation. Debates over the ethics of research into lead abatement methods, prompted by the controversy surrounding the Kennedy-Krieger Institute Lead Paint Repair and Maintenance Study, provide a helpful illustration of this problem.

The Kennedy-Krieger Institute (KKI) Lead Paint Repair and Maintenance Study recruited 108 families, including children 6 months to 7 years of age, from the inner city of Baltimore, Maryland, for a study of different methods of environmental lead reduction in older homes (Nelson 2002). The study was designed to test the short-term and long-term efficacy of partial or interim lead abatement measures by comparing lead levels in homes treated by those measures against previously treated and new homes. Two sets of parents brought suit against KKI, arguing that they had not been warned about the risks to their children posed by existing or remaining levels of lead in partially or previously abated homes. The Maryland Court of Appeals overturned a summary judgment issued to the defendants, charging that the parents had not been sufficiently informed of the purpose of the research and that otherwise healthy children were treated as “canaries in the mines” (Nelson 2002).

Debates over the ethics of the KKI study have focused rightly on what level of risk is appropriate in nontherapeutic research involving children and the nature and extent of informed consent in research involving vulnerable populations, in this case, low-income African-American families living in the inner city (Bellinger and Dietrich 2002; Needleman 2002; Nelson 2002; Pinder 2002). It is beyond the scope of this commentary to treat the complex ethical and legal issues at stake in this case or to judge whether or not the criticisms of the research design or its conduct are valid. For these purposes, the interesting point of controversy concerns the relationship of studies aimed at bringing about feasible, incremental improvements in the overall environmental health of children to the goals of prevention of risk and promotion of environmental health for all children. In other words, the difficult question raised by the KKI study is how to think about the meaning of “pragmatic solidarity” under conditions where there exist insufficient political will or economic means to address the underlying conditions that place children at risk—in this case, ambient risks of lead exposure in the housing options for low-income Americans.

Buchanan and Miller (2006), among others, have defended the KKI study on the grounds that it sought to give solid evidence for evaluating feasible solutions to the problem of housing-related lead exposure in the absence of evidence that ideal solutions would be developed or implemented. As Bellinger and Dietrich (2002) put it, “With the best of intentions, the Kennedy-Krieger researchers were attempting to identify economical but effective solutions to a problem for which the political will to pursue a lasting solution is, as it has always been, sorely lacking.” In this view, the question of fairness in research must be posed within realistic considerations of the likelihood of universal implementation of public health goals. If complete lead abatement is likely to occur, there would be no ethical rationale for testing partial, less effective methods. However, the KKI study took as its premise the need, in the absence of a material social and political commitment to universal safe housing, to develop lower-cost methods for achieving incremental gains in reducing lead exposure. Public health research aimed at developing less expensive yet less effective interventions can be justified according to this position under four conditions: *a*) there is a large population in need; *b*) there exists a more effective but significantly higher cost standard of treatment and a lower cost standard of treatment that is still hypothesized to be effective; *c*) resource or political constraints do not allow full or extensive provision of the higher standard; and *d*) there exists a high degree of likelihood that the less costly intervention can and will be implemented on a wide scale (Buchanan and Miller 2006).

Although the obligations to protect human subjects from exploitation or harm within research continue to constrain the conduct of research, this position holds that it is not inherently exploitive to employ a different standard of care under such conditions: “From a public health standpoint, if it is not feasible to extend the current standard of care to the population as a whole, the appropriate control group with respect to the question of inequitable treatment is those who do not now have access to the current standard of care” (Buchanan and Miller 2006). In this sense, the relevant analogy for the KKI study was not the Tuskegee syphilis study, as the Maryland Court invoked, but studies such as those conducted in developing countries to test the efficacy of a short course of AZT in reducing maternal–child transmission of HIV in areas where gold-standard protocols are unaffordable. There, also, the controversial ethical questions concerned what should be considered therapeutic benefit for purposes of justifying the use of a lesser standard of care than is available in developed countries and whether the effort to develop feasible and affordable but less effective strategies for reducing HIV infection in newborns constituted an unacceptable double standard (de Zulueta 2001). In both cases, defenders argued that the failure to conduct such research constitutes greater harm by depriving disadvantaged populations of the benefits of achievable albeit less than ideal improvements in health (Buchanan and Miller 2006).

Farmer (1999) argues that defending a thorough-going pragmatic approach to the ethics of public health research takes systemic injustice as an unavoidable reality and risks substituting the values of efficiency and efficacy for commitments to equity. For this position, political realism is not just a qualifier to idealism but its enemy. To accept the need to develop methods for low-cost partial lead abatement falls short of a social commitment to the only effective means of protecting children from exposure to housing-related lead (i.e., providing universal access to safe housing options), but may further undermine already insufficient political will to enact reform (e.g., through addressing the connections among health, race, and poverty). Although it is “difficult, perhaps impossible, to meet the highest standards of heath care in every situation... projects striving for excellence—rather than, say, ‘cost-efficacy’ or ‘sustainability’” are not merely ‘misguided quests’” but the only acceptable ethical response to demands by the most vulnerable for justice (Farmer 1999).

Both positions raise important questions about the relationship between long-range goals and short-term strategies to benefit persons or groups at immediate risk; both raise without resolving the question of whether different contexts generate different moral standards; and both suggest that deeper issues of social, economic, and environmental justice cannot be detached from judgments about the ethical conduct of environmental health research, even if those issues have different weight for each. Yet neither suggests how exactly to resolve apparent conflicts between the goals of broad social transformation and the immediate needs of vulnerable persons, or how to reconcile ideals and realities in choices about research goals and in decisions about health policy. These issues are a subset of the larger question raised above—of the relationship between public health research and advocacy. Although a human rights approach makes clear that ethical research in pediatric environmental health is in some sense advocacy, the precise meaning of “advocacy” in the varied contexts in which that research will be proposed, conducted, and evaluated remains to be developed.

## Conclusion

A human rights paradigm for pediatric environmental health research assumes the importance of efforts to extend protection from environmental health risks to all persons and communities. Because it aims at problem solving and capacity building, it incorporates support for community-based participatory research and takes seriously the social responsibilities of researchers in efforts to achieve equity. In so doing, a human rights approach shows promise for addressing some of the unique issues that arise in the context of pediatric environmental health. At the same time, challenges remain in developing a human rights framework and applying it to environmental health research, particularly how to understand the relationship between the pursuit of ethical ideals and a commitment to pragmatic solidarity.
